# Perceptions and Acceptance of mHealth in Patients With Cardiovascular Diseases: A Cross-Sectional Study

**DOI:** 10.2196/10117

**Published:** 2019-02-04

**Authors:** Jie Jiang, Qin Zhu, Yimei Zheng, Yajing Zhu, Yuxi Li, Yong Huo

**Affiliations:** 1 Department of Cardiology Peking University First Hospital Beijing China; 2 Health Systems Philips Research China Shanghai China

**Keywords:** age, cardiovascular disease, internet, mHealth, mobile phone, secondary prevention, self-management

## Abstract

**Background:**

Mobile health (mHealth)—a method of assisting long-term care in patients with chronic cardiovascular diseases (CVDs)—is gaining popularity in China, mainly owing to the large number of patients and limited clinical resources. Patients of different ages have varying needs for CVD management. However, evidence regarding how age influences Chinese CVD patients’ use and perceptions of mHealth is limited.

**Objective:**

This study aimed to explore age-related differences among Chinese patients with CVD regarding their use and perceptions of mHealth and to determine the factors that influence this population’s willingness to use mHealth technologies.

**Methods:**

We conducted a cross-sectional study of patients with chronic CVDs in a tertiary hospital in Beijing using a new questionnaire designed by the investigators. Participants were sourced using nonproportional quota-sampling methods, being recruited consecutively in each sampling category (age 18-49, 50-64, 65-74, and ≥75 years, with at least 25 men and 25 women in each age group). The survey consisted of 5 parts, including sociodemographic profile and medical history; current disease management situation; self-evaluation of disease management; current usage of mobile and internet technology (IT); and willingness to use an mHealth solution to perform disease self-management. Responses were compared among the 4 age groups as well as between patients who were willing to use mHealth solutions and those who were not. Multivariate logistic regression model was used to identify predictors of willingness to use mHealth for self-management.

**Results:**

Overall, 231 patients (124 men) completed the questionnaire; of these, 53 were aged 18-49 years, 66 were aged 50-64 years, 54 were aged 65-74 years, and 58 were aged ≥75 years. Patients in the older cohorts visited hospitals more often than did those in the younger cohorts (*P*<.001), and they also showed lower technology skills regarding the use of mobile or internet devices (*P*<.001) and searched for health-related information on the internet less often (*P*<.001). In addition, 68.0% (157/231) of the patients showed interest in using mHealth solution to manage their disease; of these, 40.8% (64/157) were aged ≥65 years. Patients who were more willing to use mHealth solution to manage their diseases were younger (*P*<.001), more educated (*P*<.001), still working (*P*=.001), possessed higher skill regarding mobile or internet device use (*P*<.001), and more frequently searched for health information on the internet (*P*<.001). Finally, multivariate logistic regression showed that IT skill was the single indicator (*P*=.003) of willingness to use mHealth, not age.

**Conclusions:**

Although age is associated with the use of mobile or internet devices, the sole indicator of mHealth use for self-management was participants’ IT skills. Education regarding the use of mobile devices and development of easy-to-use software might improve the acceptance of mHealth solutions among older patient populations.

## Introduction

Cardiovascular disease (CVD) is a major cause of death and disability worldwide, and its prevalence is increasing, particularly in low- and middle-income countries [1]. Approximately 290 million people in China have CVD, and this number is projected to grow rapidly over the coming decade [2]. Secondary prevention of CVD is critical for improving health outcomes and quality of life, as well as for achieving cost-effective health care [1]; however, for patients with CVD, the adherence rate to secondary prevention is relatively low. For example, a meta-analysis of 376,162 patients showed that the overall adherence to drugs that prevent CVD is just 57% [3]. The magnitude and impact of poor adherence in developing countries, like China, are assumed to be even higher given the paucity of health resources and existing inequities concerning access to health care [4]. This is important because poor adherence in chronic patients has been directly correlated with a greater incidence of recurrent cardiovascular events and increases in direct and indirect health care costs [1].

There is considerable international interest in mobile health (mHealth) as a possible intervention for people with chronic diseases, including patients with CVD [5]. Previous studies demonstrated that the application of mHealth technologies among patients with CVD has significant positive effects on self-care awareness, confidence regarding disease management, adherence behavior, and clinical outcomes. In addition, it has been indicated that mHealth-based self-management programs might help patients actively control and improve their disease condition [6]. Studies have shown that a daily automated short message service text message reminder can effectively increase medication adherence among patients with acute coronary syndrome during the early postdischarge period [7,8]. After using an mHealth system consisting of an automatic sphygmomanometer, lipid meter, and mobile phone, the intervention group of acute coronary syndrome survivors showed lower body mass index and had more patients achieving treatment goals for blood pressure (BP) and hemoglobin A_1c_ than did the usual care group at 12-month follow-up [9]. Individuals with a parental history of dementia showed better adherence and decreased BP level after using mobile phone-assisted technology during a 6-month period [10]. An mHealth self-monitoring program motivated patients with hypertension to improve their health management and led to decreased cigarette smoking, alcohol use, and BP level after 6 months [11]. Thus, mHealth technologies for the secondary prevention of CVD have the potential to improve the psychological condition, medication adherence, clinical outcome, and finally the lives of patients.

Success in designing mHealth interventions requires an adequate understanding of the patients’ level of acceptance of the relevant technologies. Although diagnoses of CVD in younger populations are becoming more common, the main patient population remains those aged >65 years. Age at the time of diagnosis is a meaningful factor for long-term disease management. Younger patients are typically still working and may not pay enough attention to their disease, even though they will live with the disease and treatment for a longer time. On the other hand, the elderly take their disease and health more seriously but may lack the ability to perform self-management. Ensuring appropriate intervention designs for an audience of diverse ages and life stages is important, given these considerations.

Although many studies have been conducted on the topic of mHealth in the CVD population, most are in the Western context. A small number of studies have been conducted among the population of China, but these have been limited to narrowly defined interventions [12]. Furthermore, although most studies consider age to be a meaningful factor for evaluating acceptance and willingness, few have considered age-related differences concerning the use and perceptions in this regard.

Thus, to deepen the understanding of mHealth perceptions and acceptance, this study aimed to explore the use and perceptions of mHealth among Chinese patients with CVD using age-specific cohorts and to examine the factors that may influence this population’s willingness to use mHealth technologies.

## Methods

### Study Design and Sample Strategy

This study was conducted in the Cardiology Department of Peking University First Hospital (PKU1), which is a tertiary hospital in the city of Beijing, China. This study was approved by the Clinical Research Ethical Committee of PKU1. All respondents provided written informed consent before the study questionnaires were distributed.

Recruitment was conducted in 2016 in the Cardiology Department of PKU1, including both inpatients and outpatients, following a cross-sectional study design. These participants were sourced using nonproportional quota-sampling methods. Patients were recruited consecutively in each sampling category based on age and gender; the minimum number of sampled units in each category was 25.

The inclusion criteria were as follows: (1) patients aged >18 years; (2) those capable of providing informed consent; (3) those being inpatients or outpatients of the Cardiology Department; and (4) those requiring long-term medication therapy because of hypertension, coronary heart disease, heart failure, or arrhythmia.

### Instrumentation

To evaluate the applicability and perceptions of CVD-related mHealth, a survey method was used. A paper-and-pencil questionnaire was designed that featured multiple types of response scales with closed-ended questions. The survey was divided into 5 parts as follows: (1) sociodemographic profile and medical history; (2) current situation of disease management; (3) self-evaluation for disease management; (4) current usage of mobile and internet technology (IT); and (5) willingness to use a telehealth solution with a self-management system. The full questionnaire, with measurement responses, is displayed in Multimedia Appendix 1.

We developed the questionnaire based on a review of related studies [12,13], which was then evaluated by 3 cardiologists and 3 cardiology nurses. Next, the questionnaire was pretested in 10 patients, and minor adjustments were made depending on their responses.

### Data Collection

Data were collected during the interviews with patients, which were conducted by several research nurses, using a case record form. All research nurses were trained by one of the authors to ensure they understood the questionnaire equally. A nurse reviewed the questionnaire immediately after a patient had finished it to confirm the completeness of the questionnaire.

Two people with experience in data entry independently entered all case record form data into separate EpiData (Version 3.1, EpiData Software, Odense, Denmark) databases. The 2 databases were compared, and discrepancies were resolved by checking the original questionnaire.

### Statistical Analysis

We used descriptive statistics and chi-square statistics to present the sociodemographic and CVD characteristics. Frequencies and percentages were evaluated for categorical variables, and means and SD were determined for continuous variables. For missing data, no imputation was performed during the analysis, and the actual percentages were used for describing categorical variables. In this study, age was stratified into 4 groups as follows: 18-49 years; 50-64 years; 65-75 years; and ≥75 years. In addition, 4 groups of questions were used to create a combined score to illustrate the overall impression on the responses for each group. “Frequency of visiting hospitals” was a combined score of “frequency of visiting hospital clinic” and “frequency of visiting community clinic”; “self-management at home” was a combined score of “frequency of measuring blood pressure at home,” “recording blood pressure at home,” “using pill box at home,” and “using wearable devices as self-management at home”; “self-management confidence” was a combined score of “capable of controlling one’s health condition,” “managing one’s health behavior,” “care for oneself at home,” and “have adequate knowledge to be able to care for oneself”; finally, “IT skill” score was created from the score for using smartphone, tablet, and personal computer (PC), as well as the capability to use Wi-Fi and install apps. For each question included in the combined scores, a score of 1-5 was given to each of the original responses. If there were 5 stage responses, they were given a 1-5 sequentially; for example, *Never*=1, *1-3 per year*=2, *1-3 per month*=3, *1-3 per week*=4, and *Everyday*=5. If there were binary responses, a 1 was given to *no* responses, while a 5 was given to *yes* responses. The sum of the scores for each question formed the combined results.

Chi-square statistics and analysis of variance were performed to test whether there were associations of age with (1) the current situation of disease management, (2) self-evaluation of disease self-management, and (3) current usage of mobile or IT technology as well as to examine the associations between the willingness to apply mHealth solutions and other variables. In addition, a multivariate logistic regression analysis was performed to identify the indicators of the willingness to use mHealth to manage CVD. Gender and variables with *P*<.10 were included in the regression analysis. We regarded *P*<.05 as statistically significant. All analyses were conducted using RStudio (Version 0.99.903, 2009-2016 RStudio, Inc)

## Results

### Sociodemographic and Medical History

Overall, 231 patients with CVD were recruited and completed the survey. The patients’ sociodemographic and main medical history are summarized in Table 1. Mean body mass index was 26.7 (SD 4.6) kg/m^2^, which was similar across the 4 age groups. Almost half (103/229, 45.0%) of the patients had a university or master’s degree, and the education level significantly differed within the 4 age groups (*P*<.001), with 73.1% (38/53) of patients in the 18-49 years group having a university or master’s degree, while this percentage was only 32%-44% in other groups. Meanwhile, 64.5% (147/228) of patients were retired, including all but 1 patient in the group over 64 years and 56.2% (36/66) of participants in the 50-64 years age group. Only 10.4% (24/230) of participants were living outside of Beijing, and these were mostly younger patients (10/53, 18.9%, and 11/66, 16.7%, in the 18-49 and 50-64 years age groups, respectively). More than half of the patients (157/229, 68.6%) reported that they lived with their spouse. Almost half of the patients (131/231, 56.7%) were diagnosed with coronary artery diseases, mostly in the 3 older groups. Only 32.1% (17/53) of patients in the 18-49 years group had coronary artery diseases. Meanwhile, the percentage or hypertension in this younger group was 69.8% (37/53), which was higher than the average (120/231, 51.9%).

### Current Usage of Mobile and Internet Technologies

Overall, 67.4% (155/230) of participants reported that they used smartphone every day, while almost half had never used a tablet (52.2%, 119/228) or PC (48.0%, 109/227). Meanwhile, 69.9% (160/229) reported that they were able to connect to Wi-Fi using their smartphone or tablet. However, 60.5% (138/228) reported that they did not know how to install a new app on their smartphones or tablets. Table 2 displays patients’ current usage conditions of mobile or IT and its association with age. The older cohort used smartphones, tablets, and PCs less frequently than did the younger cohort (*P*<.001). Similarly, a smaller proportion of older patients were able to connect to Wi-Fi and install apps (*P*<.001).

**Table 1:** Sociodemographic and medical history of patients by age.

Characteristics			Total (N=231), n (%)	Age stratification in years, n (%)				Chi-square test	
				18-49 (n=53)	50-64 (n=66)	65-74 (n=54)	≥75 (n=58)	*χ^2^ (df^a^)*	*P* value
**Gender, n (%)**								1.2 (*3*)	.76
	Male		124 (53.7)	28 (52.8)	39 (59.1)	28 (51.9)	29 (50.0)		
	Female		107 (46.3)	25 (47.2)	27 (40.9)	26 (48.1)	29 (50.0)		
**Body mass index stratification in kg/m^2^, n (%)**								4.6 (*3*)	.21
	<24		61 (27.2)	13 (26.5)	12 (18.5)	16 (30.2)	20 (35.1)		
	≥24		163 (72.8)	36 (73.5)	53 (81.5)	37 (69.8)	37 (64.9)		
**Education level, n (%)**								25.9 (*6*)	<.001
	Primary or junior		67 (29.3)	9 (17.3)	20 (30.3)	19 (35.2)	19 (33.3)		
	Senior		59 (25.8)	5 (9.6)	25 (37.9)	16 (29.6)	13 (22.8)		
	University or postgraduate		103 (45.0)	38 (73.1)	21 (31.8)	19 (35.2)	25 (43.9)		
**Employment status, n (%)**								153.2 (*6*)	<.001
	Retired		147 (64.5)	1 (1.9)	36 (56.2)	52 (98.1)	58 (100.0)		
	Part time		21 (9.2)	11 (20.8)	9 (14.1)	1 (1.9)	0 (0.0)		
	Full time		60 (26.3)	41 (77.4)	19 (29.7)	0 (0.0)	0 (0.0)		
**Home location, n (%)**								14.0 (*3*)	.003
	Beijing		206 (89.6)	43 (81.1)	55 (83.3)	52 (96.3)	56 (98.2)		
	Outside of Beijing		24 (10.4)	10 (18.9)	11 (16.7)	2 (3.7)	1 (1.8)		
**Living status, n (%)**								7.0 (*6*)	.32
	Alone		14 (6.1)	3 (5.9)	1 (1.5)	5 (9.3)	5 (8.6)		
	Only with spouse		157 (68.6)	35 (68.6)	52 (78.8)	35 (64.8)	35 (60.3)		
	With children		58 (25.3)	13 (25.5)	13 (19.7)	14 (25.9)	18 (31.0)		
**Enrollment location**								32.1 (*3*)	<.001
	Outpatient		145(61.7)	37(69.8)	20(29.9)	16(28.6)	17(28.8)		
	Inpatient		90(38.3)	16(30.2)	47(70.1)	40(71.4)	42(71.2)		
**Medical history**									
	**Coronary heart disease, n (%)**							25.0 (*3*)	<.001
		No	100 (43.3)	36 (67.9)	22 (33.3)	13 (24.1)	29 (50.0)		
		Yes	131 (56.7)	17 (32.1)	44 (66.7)	41 (75.9)	29 (50.0)		
	**Hypertension, n (%)**							12.7 (*3*)	.005
		No	111 (48.1)	16 (30.2)	37 (56.1)	33 (61.1)	25 (43.1)		
		Yes	120 (51.9)	37 (69.8)	29 (43.9)	21 (38.9)	33 (56.9)		
	**Arrhythmia, n (%)**							10.8 (*3*)	.01
		No	169 (73.2)	44 (83.0)	53 (80.3)	38 (70.4)	34 (58.6)		
		Yes	62 (26.8)	9 (17.0)	13 (19.7)	16 (29.6)	24 (41.4)		
	**Heart failure, n (%)**							5.9 (*3*)	.12
		No	212 (91.8)	47 (88.7)	64 (97.0)	51 (94.4)	50 (86.2)		
		Yes	19 (8.2)	6 (11.3)	2 (3.0)	3 (5.6)	8 (13.8)		
	**Diabetes, n (%)**							5.2 (*3*)	.16
		No	189 (81.8)	40 (75.5)	51 (77.3)	46 (85.2)	52 (89.7)		
		Yes	42 (18.2)	13 (24.5)	15 (22.7)	8 (14.8)	6 (10.3)		
	**Hyperlipidemia, n (%)**							4.3 (*3*)	.23
		No	156 (67.5)	33 (62.3)	40 (60.6)	40 (74.1)	43 (74.1)		
		Yes	75 (32.5)	20 (37.7)	26 (39.4)	14 (25.9)	15 (25.9)		
	**Cerebrovascular disease, n (%)**							3.4 (*3*)	.34
		No	214 (92.6)	52 (98.1)	61 (92.4)	49 (90.7)	52 (89.7)		
		Yes	17 (7.4)	1 (1.9)	5 (7.6)	5 (9.3)	6 (10.3)		
	**Peripheral vascular disease, n (%)**							2.9 (*3*)	.41
		No	211 (91.3)	51 (96.2)	58 (87.9)	50 (92.6)	52 (89.7)		
		Yes	20 (8.7)	2 (3.8)	8 (12.1)	4 (7.4)	6 (10.3)		

^a^*df*: degrees of freedom.

### Current Disease Management

The frequency of searching for health information using the internet was found to be significantly correlated with age (*P*<.001); approximately 52.8% (94/178) of patients aged >50 years never searched the health information on the internet, while this percentage was only 11.3% (6/53) of the youngest group. Meanwhile, 78.9% (179/227) of patients reported that they were familiar with the medication they were taking. The frequency of visiting hospital clinics showed a significant difference across the 4 age groups (*P*=.004); >66.1% (74/112) of patients aged >65 years visited hospitals monthly. Although it seems that more patients aged ≥75 years used a pill box, there was no statistically significant difference among groups in this regard. In addition, the combined score for self-management at home had no significant correlation with age (*P*=.28). The participants’ situation regarding disease management in terms of age is displayed in Table 3.

### Self-Evaluation of Disease Management

Most patients reported being capable of controlling their health conditions (146/223, 65.5%), managing their health behaviors (150/222, 67.6%), and caring for themselves at home (175/221, 79.2%), but only 57.0% (126/221) believed that they had adequate knowledge to be able to care for themselves. In addition, the combined score of “self-management confidence” had no significant association with age (*P*=.60). Although no significant associations between self-evaluation of disease management and age were found, numerically patients aged <50 years and ≥75 years were less confident in their knowledge of their disease. Table 4 displays the self-evaluation of disease management and its association with age concerning our respondents.

### Willingness to Use an mHealth Solution With a Self-Management System

Overall, 68.0% (157/231) of the participants were willing to use mHealth technologies to manage their CVD, of which 63.2% (86/136) felt that it might benefit them and 26.5% (36/136) patients reported just wanting to try it. Of the 32.0% (74/231) who reported that they were not interested in an mHealth solution, the major reasons were that they were unable to use the devices (64.4%, 47/73) and that the devices were too complicated for them (16.4%, 12/73). Participants were divided into 2 groups according to their willingness to use mHealth technologies. Patients in the younger cohort, who had a higher education level, who were still working, and had higher IT skills, had a higher frequency of internet searching, had the habit of self-management at home, and showed more interest in mHealth solution. The associations between willingness and other variables are shown in Table 5.

Multivariate logistic regression showed that although the willingness to use an mHealth solution varied among different age groups, this difference vanished after performing the multivariate logistic regression. Consequently, the IT skill was determined to be the only independent indicator for the willingness to use an mHealth solutions (*P*=.02). Results of multivariate logistic regression analysis are summarized in Table 6.

**Table 2:** Current usage of mobile or internet technology (IT) among patients and its association with age.

Characteristics		Total (N=231), n (%)	Age stratification in years, n (%)				Chi-square test		
			18-49 (n=53)	50-64 (n=66)	65-74 (n=54)	≥75 (n=58)	*χ^2^ (df*^a^)		*P* value
**Smartphone, n (%)**							57.0 (*12*)	<.001	
	Never	55 (23.9)	2 (3.8)	8 (12.1)	19 (35.2)	26 (44.8)			
	1-3 per year	3 (1.3)	0 (0.0)	2 (3.0)	0 (0.0)	1 (1.7)			
	1-3 per month	5 (2.2)	0 (0.0)	2 (3.0)	2 (3.7)	1 (1.7)			
	1-3 per week	12 (5.2)	1 (1.9)	0 (0.0)	4 (7.4)	7 (12.1)			
	Everyday	155 (67.4)	49 (94.2)	54 (81.8)	29 (53.7)	23 (39.7)			
**Tablet, n (%)**							46.0 (*12*)	<.001	
	Never	119 (52.2)	10 (18.9)	37 (56.9)	32 (60.4)	40 (70.2)			
	1-3 per year	6 (2.6)	3 (5.7)	0 (0.0)	0 (0.0)	3 (5.3)			
	1-3 per month	10 (4.4)	7 (13.2)	1 (1.5)	1 (1.9)	1 (1.8)			
	1-3 per week	29 (12.7)	12 (22.6)	8 (12.3)	6 (11.3)	3 (5.3)			
	Everyday	64 (28.1)	21 (39.6)	19 (29.2)	14 (26.4)	10 (17.5)			
**Personal computer, n (%)**							45.2 (*12*)	<.001	
	Never	109 (48.0)	10 (18.9)	27 (41.5)	34 (65.4)	38 (66.7)			
	1-3 per year	10 (4.4)	3 (5.7)	3 (4.6)	2 (3.8)	2 (3.5)			
	1-3 per month	8 (3.5)	2 (3.8)	1 (1.5)	3 (5.8)	2 (3.5)			
	1-3 per week	20 (8.8)	3 (5.7)	9 (13.8)	4 (7.7)	4 (7.0)			
	Everyday	80 (35.2)	35 (66.0)	25 (38.5)	9 (17.3)	11 (19.3)			
**Able to connect to Wi-Fi, n (%)**							25.0 (*3*)	<.001	
	No	69 (30.1)	5 (9.4)	15 (22.7)	20 (38.5)	29 (50.0)			
	Yes	160 (69.9)	48 (90.6)	51 (77.3)	32 (61.5)	29 (50.0)			
**Able to install apps, n (%)**							60.8 (*3*)	<.001	
	No	138 (60.5)	10 (18.9)	39 (59.1)	39 (75.0)	50 (87.7)			
	Yes	90 (39.5)	43 (81.1)	27 (40.9)	13 (25.0)	7 (12.3)			
IT skills^b^ (score), mean (SD)		15.57 (7.16)	21.23 (4.84)	16.63 (6.38)	13.12 (6.39)	11.30 (6.78)	77.5 (*3*)^c^		<.001^c^

^a^*df*: degrees of freedom.

^b^“IT skills” was a combined result of the 5 questions in this part.

^c^The result was obtained using analysis of variance.

**Table 3:** Current situation of disease management by age.

Characteristics		Total (N=231), n (%)	Age stratification in years, n (%)				Chi-square test	
			18-49 (n=53)	50-64 (n=66)	65-74 (n=54)	≥75 (n=58)	*χ^2^ (df^a^)*	*P* value
**Frequency of using the internet to find information about disease or treatment, n (%)**							43.2 (*9*)	<.001
	Never	100 (43.5)	6 (11.3)	26 (40.0)	30 (55.6)	38 (65.5)		
	Rarely	27 (11.7)	7 (13.2)	10 (15.4)	7 (13.0)	3 (5.2)		
	Sometimes	49 (21.3)	18 (34.0)	11 (16.9)	9 (16.7)	11 (19.0)		
	Often	54 (23.5)	22 (41.5)	18 (27.7)	8 (14.8)	6 (10.3)		
**Know your medications, n (%)**							6.9 (*3*)	.08
	No	48 (21.1)	9 (17.3)	21 (32.3)	8 (15.4)	10 (17.2)		
	Yes	179 (78.9)	43 (82.7)	44 (67.7)	44 (84.6)	48 (82.8)		
**Frequency of visiting hospital clinic, n (%)**							27.9 (*12*)	.004
	Never	31 (13.6)	8 (15.4)	15 (22.7)	6 (11.3)	2 (3.5)		
	Once per year	29 (12.7)	9 (17.3)	11 (16.7)	6 (11.3)	3 (5.3)		
	1-2 per half of year	42 (18.4)	15 (28.8)	8 (12.1)	7 (13.2)	12 (21.1)		
	1-2 per month	120 (52.6)	20 (38.5)	31 (47.0)	33 (62.3)	36 (63.2)		
	Weekly	6 (2.6)	0 (0.0)	1 (1.5)	1 (1.9)	4 (7.0)		
**Frequency of visiting community clinic, n (%)**							18.1 (*12*)	.11
	Never	97 (42.5)	29 (54.7)	31 (47.7)	16 (30.2)	21 (36.8)		
	Once per year	20 (8.8)	5 (9.4)	5 (7.7)	3 (5.7)	7 (12.3)		
	1-2 per half of year	34 (14.9)	8 (15.1)	10 (15.4)	7 (13.2)	9 (15.8)		
	1-2 per month	72 (31.6)	11 (20.8)	19 (29.2)	25 (47.2)	17 (29.8)		
	Weekly	5 (2.2)	0 (0.0)	0 (0.0)	2 (3.8)	3 (5.3)		
**Frequency of taking lab test, n (%)**							18.1 (*12*)	.11
	Never	26 (11.4)	9 (17.3)	11 (16.7)	4 (7.5)	2 (3.4)		
	Once per year	63 (27.5)	16 (30.8)	20 (30.3)	11 (20.8)	16 (27.6)		
	1-2 per half of year	90 (39.3)	18 (34.6)	26 (39.4)	20 (37.7)	26 (44.8)		
	1-2 per month	45 (19.7)	9 (17.3)	7 (10.6)	17 (32.1)	12 (20.7)		
	Weekly	5 (2.2)	0 (0.0)	2 (3.0)	1 (1.9)	2 (3.4)		
**Frequency of measuring blood pressure at home, n (%)**							16.1 (*12*)	.19
	Never	40 (17.3)	8 (15.1)	15 (22.7)	12 (22.2)	5 (8.6)		
	1-3 per year	16 (6.9)	5 (9.4)	5 (7.6)	5 (9.3)	1 (1.7)		
	1-3 per month	49 (21.2)	14 (26.4)	15 (22.7)	7 (13.0)	13 (22.4)		
	1-3 per week	57 (24.7)	15 (28.3)	12 (18.2)	15 (27.8)	15 (25.9)		
	Everyday	69 (29.9)	11 (20.8)	19 (28.8)	15 (27.8)	24 (41.4)		
**Did you record your blood pressure at home, n (%)**							6.5 (*3*)	.09
	No	138 (60.3)	28 (53.8)	42 (63.6)	39 (72.2)	29 (50.9)		
	Yes	91 (39.7)	24 (46.2)	24 (36.4)	15 (27.8)	28 (49.1)		
**Did you use pill box at home, n (%)**							3.5 (*3*)	.32
	No	91 (39.6)	23 (43.4)	29 (43.9)	22 (41.5)	17 (29.3)		
	Yes	139 (60.4)	30 (56.6)	37 (56.1)	31 (58.5)	41 (70.7)		
**Did you use wearables, n (%)**							3.5 (*3*)	.32
	No	198 (88.4)	42 (82.4)	58 (87.9)	48 (94.1)	50 (89.3)		
	Yes	26 (11.6)	9 (17.6)	8 (12.1)	3 (5.9)	6 (10.7)		
Frequency of visiting hospital (score)^b^, mean (SD)		5.58 (1.93)	4.94 (1.82)	5.12 (1.98)	6.17 (1.89)	6.14 (1.70)	17.2 (*3*)^c^	<.001^c^
Self-management at home (score)^d^, mean (SD)		10.88 (4.17)	11.10 (4.43)	10.41 (3.98)	10.00 (4.25)	12.05 (3.90)	1.2 (*3*)^c^	.28^c^

^a^*df*: degrees of freedom.

^b^"Frequency of visiting hospital" is a combined result of "Frequency of visiting hospital clinic" and "Frequency of visiting community clinic".

^c^The result was obtained using analysis of variance.

^d^"Self-management at home" was a combined result of "Frequency of measuring blood pressure at home", "Did you record your blood pressure at home", "Did you use pill box at home", and "Did you use wearables".

**Table 4:** Self-evaluation of disease management by age.

Characteristics		Total (N=231), n (%)	Age stratification in years, n (%)				Chi-square test	
			18-49 (n=53)	50-64 (n=66)	65-74 (n=54)	≥75 (n=58)	*χ^2^ (df^a^)*	*P* value
**Can control your health condition, n (%)**							5.2 (*12*)	.95
	Disagree Strongly	2 (0.9)	1 (2.0)	0 (0.0)	0 (0.0)	1 (1.7)		
	Disagree	27 (12.1)	5 (9.8)	8 (12.7)	6 (11.8)	8 (13.8)		
	Neutral	48 (21.5)	11 (21.6)	13 (20.6)	11 (21.6)	13 (22.4)		
	Agree	122 (54.7)	28 (54.9)	33 (52.4)	28 (54.9)	33 (56.9)		
	Agree Strongly	24 (10.8)	6 (11.8)	9 (14.3)	6 (11.8)	3 (5.2)		
**Can manage your health behaviors, n (%)**							9.4 (*12*)	.67
	Disagree Strongly	3 (1.4)	1 (2.0)	1 (1.6)	0 (0.0)	1 (1.8)		
	Disagree	28 (12.6)	10 (19.6)	6 (9.4)	4 (8.0)	8 (14.0)		
	Neutral	41 (18.5)	7 (13.7)	13 (20.3)	8 (16.0)	13 (22.8)		
	Agree	121 (54.5)	28 (54.9)	32 (50.0)	32 (64.0)	29 (50.9)		
	Agree Strongly	29 (13.1)	5 (9.8)	12 (18.8)	6 (12.0)	6 (10.5)		
**Can care for yourself at home, n (%)**							13.2 (*12*)	.35
	Disagree Strongly	2 (0.9)	1 (2.0)	0 (0.0)	0 (0.0)	1 (1.7)		
	Disagree	12 (5.4)	3 (5.9)	2 (3.2)	2 (4.1)	5 (8.6)		
	Neutral	32 (14.5)	8 (15.7)	7 (11.1)	4 (8.2)	13 (22.4)		
	Agree	141 (63.8)	31 (60.8)	43 (68.3)	32 (65.3)	35 (60.3)		
	Agree Strongly	34 (15.4)	8 (15.7)	11 (17.5)	11 (22.4)	4 (6.9)		
**Have enough health knowledge to be able to care for yourself, n (%)**							19.4 (*12*)	.08
	Disagree Strongly	5 (2.3)	1 (2.0)	0 (0.0)	0 (0.0)	4 (7.0)		
	Disagree	33 (14.9)	10 (19.6)	7 (11.3)	7 (13.7)	9 (15.8)		
	Neutral	57 (25.8)	15 (29.4)	18 (29.0)	8 (15.7)	16 (28.1)		
	Agree	105 (47.5)	24 (47.1)	28 (45.2)	30 (58.8)	23 (40.4)		
	Agree Strongly	21 (9.5)	1 (2.0)	9 (14.5)	6 (11.8)	5 (8.8)		
Confidence in self-management (score)^b^, mean (SD)		14.61 (2.90)	14.25 (3.08)	15.02 (2.60)	15.22 (2.53)	13.96 (3.21)	0.3 (*3*)^c^	.60^c^

^a^*df*: degrees of freedom.

^b^Confidence in self-management is a combined result for the 4 questions in this part.

^c^The result was obtained using analysis of variance.

**Table 5:** Willingness of participants to use mobile health (mHealth) technologies to manage their cardiovascular diseases.

Characteristics		Total (N=231), n (%)	Willingness to use mHealth, n (%)		Chi-square test	
			Interest (n=157)	No interest (n=74)	*χ^2^ (df^a^)*	*P* value
Age in years, mean (SD)		61.07 (15.62)	58.29 (16.19)	66.99 (12.51)	16.7 (*3*)^b^	<.001^b^
**Age stratification in** **years,** **n (%)**					13.8 (*1*)	.003
	18-49	53 (22.9)	45 (28.7)	8 (10.8)		
	50-64	66 (28.6)	48 (30.6)	18 (24.3)		
	65-74	54 (23.4)	30 (19.1)	24 (32.4)		
	≥75	58 (25.1)	34 (21.7)	24 (32.4)		
**Gender, n (%)**					0.1 (*1*)	.73
	Male	124 (53.7)	86 (54.8)	38 (51.4)		
	Female	107 (46.3)	71 (45.2)	36 (48.6)		
**Body mass index stratification in** **kg/m^**2**^,** **n (%)**					0.00 (*1*)	.96
	≤24	61 (27.2)	41 (26.8)	20 (28.2)		
	>24	163 (72.8)	112 (73.2)	51 (71.8)		
**Education level, n (%)**					24.4 (*2*)	<.001
	Primary or junior	67 (29.3)	32 (20.5)	35 (47.9)		
	Senior	59 (25.8)	38 (24.4)	21 (28.8)		
	University or postgraduate	103 (45.0)	86 (55.1)	17 (23.3)		
**Employment status, n (%)**					13.1 (*2*)	.001
	Retired	147 (64.5)	90 (58.1)	57 (78.1)		
	Part time	21 (9.2)	13 (8.4)	8 (11.0)		
	Full time	60 (26.3)	52 (33.5)	8 (11.0)		
**Home location, n (%)**					0.00 (*1*)	>.99
	Beijing	206 (89.6)	141 (89.8)	65 (89.0)		
	Outside of Beijing	24 (10.4)	16 (10.2)	8 (11.0)		
**Living status, n (%)**					4.1 (*2*)	.13
	Alone	14 (6.1)	10 (6.5)	4 (5.4)		
	Only with spouse	157 (68.6)	112 (72.3)	45 (60.8)		
	With children	58 (25.3)	33 (21.3)	25 (33.8)		
**Enrollment location**					11.9 (*1*)	<.001
	Outpatient	87 (37.7)	71 (45.2)	16 (21.6)		
	Inpatient	144 (62.3)	86 (54.8)	58 (78.4)		
**Frequency of using the internet to find information about disease or treatment, n (%)**					36.1 (*3*)	<.001
	Never	100 (43.5)	48 (30.8)	52 (70.3)		
	Rarely	27 (11.7)	18 (11.5)	9 (12.2)		
	Sometimes	49 (21.3)	43 (27.6)	6 (8.1)		
	Often	54 (23.5)	47 (30.1)	7 (9.5)		
**Did you know the medications you took well, n (%)**					0.09 (*1*)	.77
	No	48 (21.1)	31 (20.3)	17 (23.0)		
	Yes	179 (78.9)	122 (79.7)	57 (77.0)		
**Frequency of taking lab test, n (%)**					6.1 (*4*)	.19
	Never	26 (11.4)	18 (11.5)	8 (11.0)		
	Once per year	63 (27.5)	44 (28.2)	19 (26.0)		
	1-2 per half of year	90 (39.3)	60 (38.5)	30 (41.1)		
	1-2 per month	45 (19.7)	33 (21.2)	12 (16.4)		
	Weekly	5 (2.2)	1 (0.6)	4 (5.5)		
Frequency of visiting hospital (score), mean (SD)		5.58 (1.93)	5.60 (1.88)	5.52 (2.03)	0.09 (*1*)^b^	.77^b^
Self-management at home (score), mean (SD)		10.88 (4.17)	11.24 (4.30)	10.09 (3.80)	3.7 (*1*)^b^	.05^b^
Confidence in self-management (score), mean (SD)		14.61 (2.90)	14.80 (2.77)	14.22 (3.13)	1.9 (*1*)^b^	.17^b^
Internet technology skills (score), mean (SD)		15.57 (7.16)	17.88 (6.37)	10.68 (6.24)	63.2 (*1*)^b^	<.001^b^

^a^*df*: degrees of freedom.

^b^The result was obtained using analysis of variance.

**Table 6:** Multivariate logistic regression concerning the willingness to use mobile health (mHealth) solutions.

Independent variables	Willingness to use mHealth		*P* value
	Coefficient	Odds ratio (95% CI)	
Age	–0.02	0.98 (0.94-1.02)	.35
Gender	–0.16	0.85 (0.42-1.73)	.65
Education level	0.18	1.12 (0.81-1.77)	.38
Employment status	–0.40	0.67 (0.34-1.34)	.26
Enrollment location	–0.63	0.53 (0.24-1.20)	.13
Internet technology skills	0.11	1.12 (1.03-1.21)	.006
Frequency of using the internet to search for health-related information	0.25	1.29 (0.87-1.90)	.20
Self-management at home	0.02	1.02 (0.94-1.11)	.65

## Discussion

### Principal Findings

The main findings of this study are that older patients visit hospitals more frequently than younger patients, but their frequency of searching for health-related information through the internet is lower. Older cohorts use mobile or IT less frequently and show lower technology skills than younger cohorts. More than half of the patients in the sample aged >65 years are interested in using mHealth solutions to manage their disease. Most significantly, experience in mobile technology use, not age, is the main indicator of the willingness to use mHealth.

Older patients visiting hospital more frequently showed that they have a higher motivation to manage their disease than younger patients. However, regarding their ability to manage their disease, the elderly less frequently searched for health-related information on the internet. Similar to the findings of this study, previous studies have shown that men in the older cohort (age >65 years) used the internet less frequently than younger men and also felt less comfortable using the internet [13]; meanwhile, younger patients (age <44 years) reportedly have more positive attitudes toward using mHealth apps for medication adherence than older participants [14]. However, in our research, we found that approximately 30% of our respondents aged >65 years used the internet to obtain health-related information. If a better solution for actively sending useful information to patients is developed, this may help the elderly obtain information more conveniently and improve their self-management capabilities.

As mentioned, older cohorts used the mobile or IT less frequently and showed lower technology skills than younger cohorts. These are not surprising findings. Importantly, however, a large proportion of patients used a smartphone almost every day; even in patients aged >75 years, this proportion is nearly 40%. Considering the large proportion of elderly with CVD in the population, there is huge potential for the use of smartphone apps in the self-management of CVD.

Previous studies showed that educated and younger individuals are more likely to download or use mHealth tools [12,13]. In our study, we found that the difference between older and younger patients regarding the willingness to use mHealth is based on their mobile technology proficiency, not their age. More than half of the patients aged >65 years showed an interest in using an mHealth solution. Meanwhile, patients who were unwilling to use an mHealth solution reported that this mainly related to difficulties in using IT. This is consistent with the results of previous studies that older individuals are interested in mHealth but have concerns regarding their ability to use the devices [15]. Another study also indicated that clinical staff worried that mHealth devices might be too complicated for patients to use [16]. Therefore, it is essential to make mHealth tools easier-to-use and train elderly patients in technology- related skills, which might promote their willingness to use mHealth solutions.

### Limitations

The study has several limitations. First, enrolled participants were sourced from a single tertiary hospital in Beijing, which compromises the generalizability of our results. Second, the limited sample size might constrain the power of statistical analysis. Third, based on the cross-sectional design of the study, we can only conclude that there is a significant correlation between IT skills and the willingness to use mHealth solution and cannot draw any conclusions regarding causality. Fourth, this study is based on a self-report survey of questionnaires. Using a newly designed questionnaire, even if pretested on a sample of 10 patients, does not bear the same weight as a validated questionnaire in prior studies. Thus, further validation of the instrument may be needed. Another limitation to note is that our items focused on the individuals’ “willingness” to use mHealth, so we were examining their intent to use the technology rather than their actual use of it.

### Conclusions

Although age is associated with the use of mobile or internet devices in the univariate analyses in this study, the sole indicator that we identified for mHealth use for self-management was the participants’ IT skills. Education on the skills of using mobile devices and the invention of easy-to-use software might improve the acceptance of mHealth solutions among older patient populations.

## Appendix

Multimedia Appendix 1 Survey questionnaire.

## Abbreviations

BP: blood pressure
CVD: cardiovascular disease
IT: internet technology
mHealth: mobile health
PC: personal computer
PKU1: Peking University First Hospital
