# Supplementary material for: Perceptions and Acceptance of mHealth in Patients With Cardiovascular Diseases: A Cross-Sectional Study
Source: JMIR Mhealth Uhealth. 2019 Feb 4;7(2):e10117. doi: 10.2196/10117 (PMC6483059; doi:10.2196/10117)
Supplement: Multimedia Appendix 1 [file mhealth_v7i2e10117_app1.pdf]

Appendix 1 Survey questionnaire

Age		Sex	
Hight	cm	Body weight	kg
Education	☐Primary or junior school<br>☐Senior school<br>☐University or post-graduate		
Employment status	☐Retired ☐Part-time ☐Full-time		
Home location	☐Beijing ☐Outside Beijing		
Living status	☐Living alone<br>☐Living only with husband/wife<br>☐Living with children		
Medical History	☐Coronary heart disease<br>☐Hypertension,<br>☐Arrhythmia<br>☐Heart Failure	☐Diabetes<br>☐Hyperlipidemia<br>☐Cerebrovascular disease<br>☐Peripheral vascular disease	

**Current situation of disease management**	
How often have you used the Internet to find information about your disease or treatment	☐Never ☐Rarely<br>☐Sometimes ☐Often
Do you understand the benefits and side effects of the medication you currently taking	☐No ☐Yes
How often have you used to visit hospital clinic	☐Never<br>☐Once per year<br>☐1-2 times per half of year<br>☐1-2 times per month<br>☐weekly
How often have you used to visit community clinic	☐ Never<br>☐Once per year<br>☐1-2 times per half of year<br>☐1-2 times per month<br>☐weekly
How often have you used to take lab tests	☐ Never<br>☐Once per year<br>☐1-2 times per half of year<br>☐1-2 times per month<br>☐weekly
How often have you measured your blood pressure at home	☐Never<br>☐1-3 per year<br>☐1-3 times per month<br>☐1-3 times per week<br>☐Everyday
Do you record blood pressure at home	☐No ☐Yes
Do you use pill box at home	☐No ☐Yes
Do you use wearable devices at home	☐No ☐Yes
**Evaluation of health self management**	
I feel I am able to control my health	☐Disagree Strongly

status	☐Disagree<br>☐Neutral<br>☐Agree<br>☐Agree Strongly
I feel I am able to manage my health behavior	☐Disagree Strongly<br>☐Disagree<br>☐Neutral<br>☐Agree<br>☐Agree Strongly
I feel I am able to take care of myself at home	☐Disagree Strongly<br>☐Disagree<br>☐Neutral<br>☐Agree<br>☐Agree Strongly
I feel I have enough health knowledge to take care of myself	☐Disagree Strongly<br>☐Disagree<br>☐Neutral<br>☐Agree<br>☐Agree Strongly
**Current usage of mobile internet technology**	
How often do you use smartphone	☐Never<br>☐1-3 per year<br>☐1-3 per month<br>☐1-3 per week<br>☐Nearly every day
How often do you use tablet	☐Never<br>☐1-3 per year<br>☐1-3 per month<br>☐1-3 per week<br>☐Nearly every day
How often do you use personal computer	☐Never<br>☐1-3 per year<br>☐1-3 per month<br>☐1-3 per week<br>☐Nearly every day
Are you able to connect to wifi using your mobile phone or tablet	☐No ☐Yes
Are you able to install a new app on your mobile phone or tablet	☐No ☐Yes
**Willingness to use a mHealth solution with a self-management system**	
Are you willing to use a mHealth app or solution to manage your disease	☐No ☐Yes
If Yes, why are you willing to try mHealth solution	☐I just want to try<br>☐I feel it will be helpful for my disease management<br>☐I am willing to use if it is recommended by physicians or nurses<br>☐Others
If No, why	☐I am not familiar with high technology

	☐I don’t think it is helpful for my disease management<br>☐I think it is valueless for me<br>☐I feel the solution is too complex to use<br>☐Other
